# *GATA2* mutation is associated with immune dysfunction and increased *Mycobacterium haemophilum* susceptibility in immunocompromised individuals

**DOI:** 10.1172/jci.insight.185582

**Published:** 2025-08-05

**Authors:** Ananya Gupta, Shail B. Mehta, Abhimanyu, Bruce A. Rosa, John Martin, Mushtaq Ahmed, Shyamala Thirunavukkarasu, Farheen Fatma, Gaya K. Amarasinghe, Makedonka Mitreva, Thomas C. Bailey, David B. Clifford, Shabaana A. Khader

**Affiliations:** 1 Department of Molecular Microbiology, Washington University in St. Louis, St. Louis, Missouri, USA.; 2 Department of Microbiology, University of Chicago, Chicago, Illinois, USA.; 3 Division of Pulmonary and Critical Care Medicine,; 4 Division of Infectious Diseases, Department of Internal Medicine,; 5 McDonnell Genome Institute,; 6 Department of Pathology and Immunology,; 7 Division of Infectious Diseases, Department of Medicine, and; 8 Department of Neurology, Washington University School of Medicine, St. Louis, Missouri, USA.

**Keywords:** Immunology, Infectious disease, Bacterial infections, Cellular immune response, Molecular pathology

## Abstract

Infections with nontuberculous mycobacterium (NTM) are on the rise. Here, we investigated an uncommon NTM infection, by *M*. *haemophilum* (*Mh*, *n* = 4), from a shared geographic location in the United States. All patients had underlying immunosuppressive conditions or treatments. We identified that all these individuals had a nonsynonymous mutation in *GATA2* gene, which was absent in healthy controls (HCs, *n* = 4) from the same geographic area (Missouri, USA). Whole blood from these individuals had attenuated cytokine responses to *Mh* stimulation for IL-1β, IL-6, IL-8, MIP-1α and MIP-1β, but not to phytohemagglutinin (PHA) or another NTM, *M*. *abscessus*. Impaired whole blood transcriptional responses in individuals with *GATA2* mutation included heightened Ras-homolog (Rho) guanosine triphosphate hydrolases (GTPase) and lowered TGF-β responses, among others. Our results highlight that, comparatively, *M*. *abscessus* and *Mh* elicit differential immune responses in humans. We identify a 23-gene signature that distinguished host response to *Mh* and *M*. *abscessus* and show that in vitro GATA2 siRNA knockdown indeed attenuated cytokine responses to *Mh*. Thus, we provide evidence that links *GATA2* mutation and immune dysfunction in individuals with compromised immunity to *Mh* infection in humans and outline host factors associated with the immune response of this clinically relevant NTM.

## Abstract 2

Gupta, Mehta, Abhimanyu et al provide new evidence that links *GATA2* mutation and immune dysfunction in individuals with compromised immunity to *M heamophilum* infection in humans

## Introduction

Infections caused by nontuberculous mycobacterium (NTM) are on the rise globally (1–4), and in developed countries, it outnumbers infections caused by *M*. *tuberculosis* (5). NTM infections are difficult to treat due to resistance to commonly available antibiotics as well as the prolonged duration of antibiotics required to achieve a cure (6). The environment is known to be a major reservoir for NTMs (7), in particular soil and drinking water (8, 9). Even though these organisms are ubiquitous in the environment, most immunocompetent people will never develop infections even with repeated exposures.

NTM infections are broad in scope and may occur in almost any part of the body. Pulmonary infections are most common, but skin/soft tissue, bone/joint, bloodstream, lymph node, and CNS infections are also well described (10). The most common risk factors associated with NTM disease include structural lung disease such as cystic fibrosis and bronchiectasis for pulmonary infections (11); acquired immunodeficiency states due to HIV, AIDS, and immunosuppression for skin/bone and disseminated infections (12); and genetic disorders predisposing patients to mycobacterial infections (13, 14). *Mh* is a slow-growing mycobacterium and emerging pathogen known to cause skin/soft tissue, bone/joint, and CNS infections (10, 11), and it is classified as an opportunistic pathogen. It has a wide geographic distribution, and infections have been reported in several continents (10). Similar to *M*. *marinum* and *M*. *ulcerans*, *Mh* requires special culture conditions at 30°C–32°C (15) and has a unique requirement for iron supplementation in media to grow (16, 17). Because of this, *Mh* infections are likely underdiagnosed.

*Mh* is most widely described as causing skin infections (10, 15), likely because of predilection for cooler temperatures. However, bone/joint and disseminated infections have also been reported in patients with HIV and organ transplant recipients receiving immunosuppression (10). Immunocompetent children have been noted to develop lymphadenitis (10). Finally, CNS infections have been described in HIV (18) and immunocompromised patients (19). The environment is thought to be the major reservoir for NTM, specifically water reservoirs (7, 20). Skin infections with NTMs have been reported after cosmetic or diagnostic procedures (21). In this study, we describe 4 immunocompromised CNS-infected individuals with *Mh* infection (*Mh*-infected). We comprehensively characterized these individuals’ in vitro cytokine and transcriptional responses and explored their exomes to identify potential mutations driving such phenotypes. We observed that these individuals had attenuated proinflammatory cytokine and chemokine production upon *Mh* but not *M*. *abscessus* (*Mab*) or Phytohemagglutinin (PHA) restimulation. The transcriptional responses in whole blood from *Mh*-infected individuals were attenuated upon *Mh*, but not to *Mab* stimulation, mirroring the cytokine responses. Comparatively, the *Mh-* and *Mab-*induced transcriptome in the *Mh*-infected individuals were distinct. Exome exploration identified *GATA2* mutation as a potential driver of the immune deficient phenotype. Thus, our results have provided evidence for a link between *GATA2* mutation and immune dysfunction in immunocompromised individuals that increase *Mh* susceptibility in humans.

## Results

### Clinical description and diagnosis of 4 cases with Mh infection.

The patient information and demographics are summarized in Table 1. Patient 1 was a 54-year-old male with poorly controlled diabetes mellitus, residing in rural Missouri (USA), who presented with left sided body pain. MRI of spine showed a lesion that initially diagnosed as transverse myelitis. He did not improve with treatment of transverse myelitis and ultimately underwent spinal cord biopsy. Biopsy showed granulomatous inflammation, and a single Acid-Fast^+^ organism using Fite Stain. He underwent treatment for presumptive CNS *Mh* infection. Patient 2 was a 74-year-old male (from the same town as patient 1) presented with right foot numbness. He was also found to have a thoracic spinal lesion and underwent a laminectomy with biopsy. Pathology showed necrotizing granulomatous inflammation. Tissue was sent to the Centers for Disease Control and Prevention (CDC) for confirmation by PCR which was positive for *Mh*. After his infection, the patient was diagnosed with chronic lymphocytic leukemia (CLL). Both patients were treated with antibiotics for several months for CNS *Mh* infection with clinical resolution. An independent clinical description for these 2 cases was published by Samudralwar et al. (22). Patient 3 was a 70-year-old male kidney transplant recipient on immunosuppressive drug tacrolimus and prednisone, who presented with skin lesions. Biopsy showed necrotic granulomas, and Acid Fast Bacilli (AFB) cultures were negative. Tissue sent to CDC confirmed *Mh* by PCR. Finally, Patient 4 was a 42-year-old male on immunosuppressive corticosteroids (prednisone) for idiopathic myositis. He presented with lymphadenopathy; biopsy of an inguinal node showed noncaseating granulomas. Biopsy material was positive for *Mh* by PCR. He later developed skin lesions as well, consistent with cutaneous disease. Both Patients 3 and 4 underwent successful treatment with prolonged course of antibiotics. The patients’ circulating cell numbers were within the normal range for lymphocytes, basophils, eosinophils, neutrophils, and monocytes (Table 1).

This case series highlights important risk factors for infection with *Mh* — immunosuppression, as well as exposure to a possible common environmental source. The series represents typical *Mh* infections — CNS, lymph node, and skin/soft tissue. It also highlights the difficulty in diagnosis, as in 3 cases, diagnosis was made by a high index of suspicion and PCR testing of biopsy tissue. In 1 case (Patient 1), definitive diagnosis was not achieved. The organism was not recovered from AFB cultures in any cases, despite incubation at lower temperature in appropriate iron-containing medium.

### Patients with Mh infection have attenuated Mh-specific proinflammatory cytokine responses.

**Figure 1:**
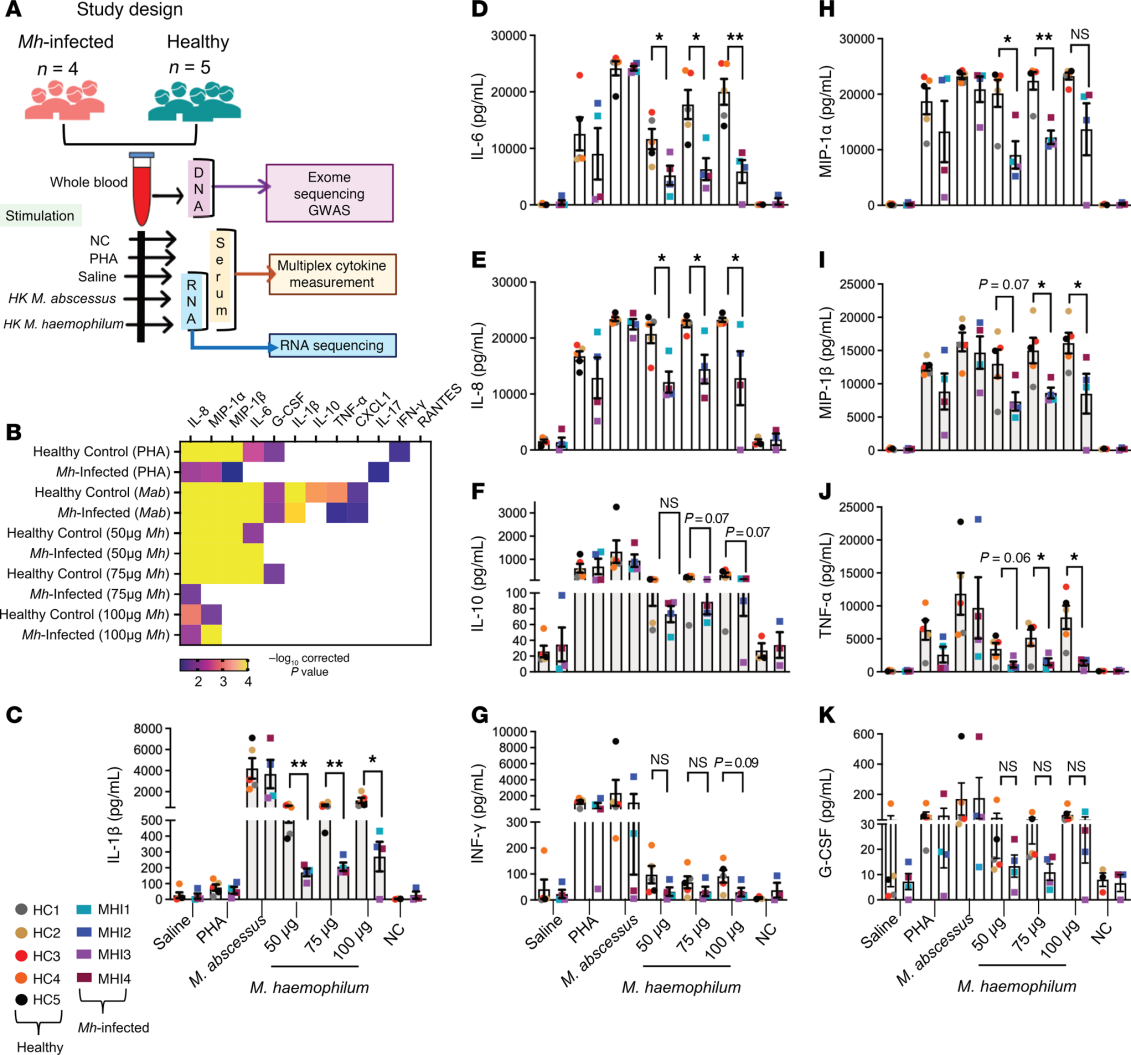
Impaired *M*. *haemophilum*–specific proinflammatory responses in individuals with *Mh* infection. (**A**) Study design schematic. (**B**) Heatmap of –log_10_ *P* value of cytokine responses to PHA, *Mab*, and *Mh* stimulations as compared with their saline controls. One-way ANOVA with Tukey’s multiple-comparison test was used. Color indicates significant comparisons. The cytokines are arranged from most to least significant in various conditions. (**C**–**K**) Specific cytokine responses to various stimulations as outlined in **A**. The cytokine levels are reported in (pg/mL) and mean ± SEM is plotted. Solid filled circles represent HCs in 4 different colors and rectangle represent the *Mh*-infected patients in 4 colors. The treatment is shown on the *x* axis; 2-tailed Student *t* test was used for the *Mh* comparisons between the HC and *Mh*-infected individuals. *P < 0.05, **P < 0.01.

To functionally characterize the immune responses in patients with *Mh*, we collected whole blood from patients and matched HCs (family members in MH1, MH2, MH3). Following 24 hours of stimulation with either heat-killed (HK) *Mh*, *Mab*, PHA, or samples left untreated (saline and negative control; only costimulatory antibodies), plasma samples were analyzed for cytokine and chemokine levels in both unstimulated and stimulated samples (Figure 1A). Upon PHA and HK *Mab* stimulation, when compared with unstimulated controls, the HCs showed potent activation and induction of cytokines including IFN-γ, G-CSF as well as chemokines such as IL-8, MIP-1α and MIP1-b; while *Mh*-infected individuals induced IL-17, and the chemokines IL-8, MIP-1α and MIP1-b (Figure 1B). Other measured chemokines GRO-α (CXCL1) and RANTES did not show any differences with PHA stimulation in either HCs or *Mh*-infected individuals. Additionally, all HCs responded to the exposure to HK *Mh* in a dose-dependent manner by inducing proinflammatory cytokines and chemokines such as IL-6, IL-8, MIP-1α, MIP-1β, and G-CSF, but not IFN-γ, as compared with the unstimulated controls (Figure 1B). Upon HK *Mh* treatment at the highest dose (100 mg), cytokine responses including IL-1β, IL-6, IL-8, TNF-α, MIP-1α, and MIP-1β were not induced to the same levels as HCs in *Mh*-infected individuals (Figure 1, C–K). Similar to HCs, no significant IFN-γ responses were induced with HK *Mh* in *Mh*-infected individuals (Figure 1, B and G). Interestingly, comparable IL-10 levels were induced upon HK *Mh* treatment in both HCs as well *Mh*-infected individuals (Figure 1F). Finally, to determine if the attenuated cytokine induction in *Mh*-infected patients was specific to treatment with HK *Mh* or was mycobacterial species specific, we treated whole blood from HCs and *Mh*-infected individuals with HK *Mab*, another NTM of clinical relevance matching the highest *Mh* dose (100 mg) (10). Indeed, both in HCs and *Mh*-infected individuals, cytokine responses including levels of TNF-α, IL-1β, IL-6, IL-8, G-CSF, IFN-γ, IL-10, MIP-1α, MIP-1β, and CXCL1 was induced upon exposure and at comparable levels in both groups of individuals (Figure 1B). These results suggest that *Mh*-infected patients can respond to exposure to other NTMs such as *Mab* and nonspecific stimuli such as mitogen (PHA) but lack the induction of *Mh*-specific immune responses. Therefore, the attenuation of cytokine responses in *Mh*-infected individuals is specific to prior exposure to *Mh*.

### Nonsynonymous SNP in GATA2 gene corroborates with high pathogenicity score in Mh patients.

**Figure 2:**
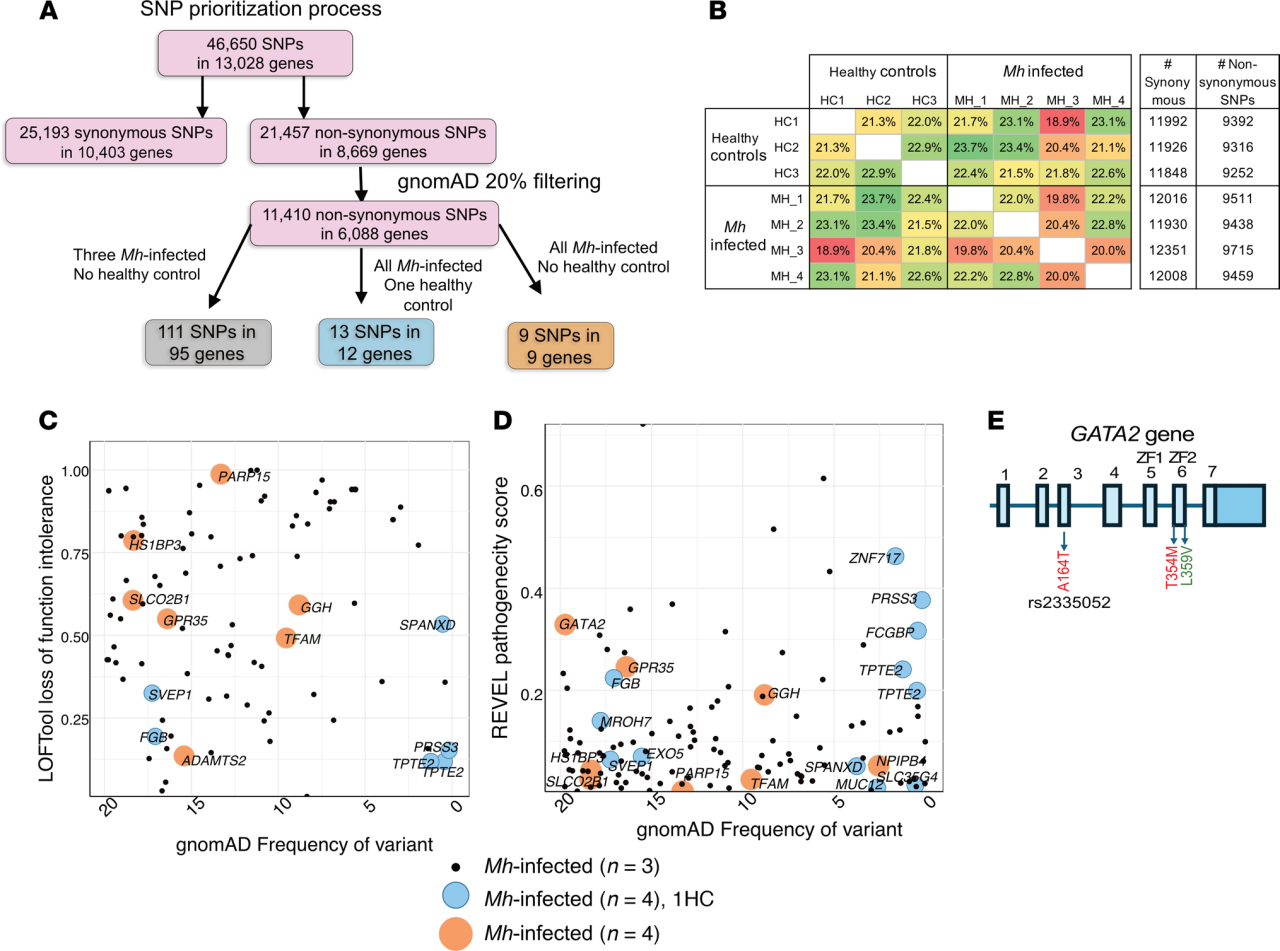
Exploring the genetic basis of impaired *M*. *haemophilum*–specific proinflammatory responses in individuals with *Mh* infection. (**A**) Schematic for single nucleotide polymorphism filtering process to identify most causal genes. (**B**) Proportion of shared synonymous and nonsynonymous SNPs between the samples of the study groups (*n* = 3 HC, and *n* = 4 *Mh*-infected). (**C**) LoFTools (114) intolerance scores, to identify gene associated with disease. (**D**) REVEL (70) score to find pathogenicity of nonsynonymous (missense) variants identified in the study. The circle size is proportional to if a HC shares the variant. Orange denotes only *Mh*-infected variants, blue has 1 HC sharing the variant, and black represent only 3 *Mh*-infected and 1 HC sharing the mutation. (**E**) The *GATA2* gene showing the exons and location of the mutant identified in this study and examples of gain-of-function (green) and loss-of-function (red) mutations.

To understand if there was a genetic basis to the *Mh*-specific lack of immune responses in *Mh*-infected individuals, we carried out whole genome sequencing of the DNA from *Mh*-infected individuals and HCs. Across 4 *Mh*-infected and 3 healthy exome sequencing samples (1 HC was excluded following analysis), 46,650 total SNPs were identified across 13,028 genes by the SNP analysis pipeline, including 25,193 synonymous SNPs and 21,457 nonsynonymous SNPs (which result in different amino acid sequences in the resulting protein) (Figure 2A). Nonsynonymous SNPs identified as being present in fewer than 20% of all individuals in the background population (according to gnomAD; ref. 23) were considered for downstream analysis, as these represent at least moderately rare variants across populations (Figure 2A). Although the overall profiles of high-impact rare SNPs showed no consistent pattern between infected individuals and HCs (Figure 2B), many of them were shared among infected samples but not among uninfected samples (Figure 2, C and D, and Supplemental Tables 2–4; supplemental material available online with this article; https://doi.org/10.1172/jci.insight.185582DS1). Using this approach, first a total of 111 high-impact SNPs in 95 genes were identified among 3 of the *Mh*-infected samples and were not identified in HC samples (Supplemental Table 2). Some of the top genes with high loss-of-function (LOF) intolerance had a high Rare Exome Variant Ensemble Learner (REVEL) pathogenicity score in the list, which included (a) *FMO2* (4 SNPs with high loss of function intolerance, LOF = 0.941, REVEL = 0.087), involved in innate immunity to TB via modulation of oxidative stress, and for which SNP variants have been associated with protective or high-risk TB progression phenotypes (24); (b) *ERAP1* (LOF = 0.999, REVEL = 0.078), in which polymorphisms are associated with *M*. *tuberculosis* infection in the Han Chinese (25); (c) *TET2* (LOF = 0.998, REVEL = 0.084), which is required for TNF promoter demethylation that drives *M*. *tuberculosis* upregulation of TNF expression in macrophages (26); (d) *SLAMF8*, which induces *M*. *tuberculosis* uptake leading to endo-lysosomal maturation in human macrophages (27) and has been identified as a biomarker of pulmonary tuberculosis, with levels associated with higher mortality and lower rates of bacterial decrease (Figure 2C and Supplemental Table 2); (e) *SELE* (*Selectin-E*) (LOF = 0.837, REVEL = 0.516), which is expressed only on the surface of activated endothelial cells, and correlates with mycobacterium bacterial load, radiological score and ESR, C-reactive protein and circulating neutrophil counts in active tuberculosis cases (28); and (f) *GZMH* (LOF = 0.358, REVEL = 0.168), which is highly expressed in NK cells and induces an alternative, caspase-independent cell-death program (29) (Figure 2D and Supplemental Table 2). Second, looking at nonsynonymous SNPS in all 4 *Mh*-infected samples and only 1 HC, an additional 13 SNPs in 12 genes were identified (Supplemental Table 3). Among these, 9 SNPs in 8 genes were identified among all 4 *Mh*-infected samples and only 1 of the HCs (Supplemental Table 3). Several of the SNPs detected in 4 *Mh*-infected samples and 1 HC were among the rarest SNPs with high REVEL pathogenicity scores, including *FCGBP* (Figure2D). This included rarer variants in (a) *FCGBP*, which is highly abundant in mucus and related to gel-forming mucins in terms of structure and localization (30), and (b) *MUC12*, a highly upregulated gene in the blood of patients with pulmonary NTM cases, compared with control subjects (31) (Figure 2D and Supplemental Table 2). Finally, 9 rare nonsynonymous mutations in 9 genes were identified in all 4 *Mh*-infected samples and none of the 3 HC samples (Supplemental Table 4 and Figure 2, C and D). Seven of these SNPs showed a high LOF intolerance score; *PARP15* showed the highest LOF intolerance score, with gnomAD frequency around 13%, followed by *HS1BPB*, *SLCO2B1*, *GPR35*, *GGH*, *TFAM* and *ADAMTS2* (Figure 2C). Several of these genes have important immunoregulatory functions; for instance, *PARP15* is a member of ADP-ribosylation signaling pathway, which has been shown to be important for regulation of infection (32) including of tuberculosis (33); *HS1BP3* induces cell apoptosis (34) and is a negative regulator of autophagy (35); *SLCO2B1* is important for maintaining iron levels and heme transport (36); *GPR35* is highly expressed in epithelial cells and important for sensing *Bacteroides fragilis* toxin and outcome of inflammatory bowel disease (37); and *TFAM* is a gene in macrophages that was found to play an important role in *M*. *bovis*–induced IFN-β production by regulating mtDNA copy numbers (38). Considering the REVEL pathogenicity score, nonsynonymous SNPs *GATA2* had the highest REVEL pathogenicity score, followed by *GPR35*, among SNPs present in all 4 *Mh*-infected individuals and no HC (Figure 2, D and E). *GATA2* is an important myeloid lineage transcription factor, mutations in which have been associated with the autosomal dominant and sporadic monocytopenia and mycobacterial infection (MonoMAC) syndrome (39). *GATA2* germline mutations can manifest as activating or gain-of-function (GOF) variants that enhance GATA2 activity, or as LOF mutation that repress GATA2 activity. Examples of GOF and LOF mutations occurring in the zinc finger domain (ZF2) include L359V, found in chronic myelogenous leukemia (40), and T354M in acute myeloid leukemia (AML) or myelodysplastic syndrome (41), respectively. A whole compendium of these mutations has been identified in various disease conditions such as AML (44 mutations) and have been described in various diseases by Collin et al. (42). Here, we describe that *GATA2* mutations (Figure 2E) in immunocompromised individuals enhanced their susceptibility to *Mh*, which could manifest in an infection of the skin and CNS.

### Individuals with Mh infection exhibit altered immune signaling pathways upon Mh reexposure.

**Figure 3:**
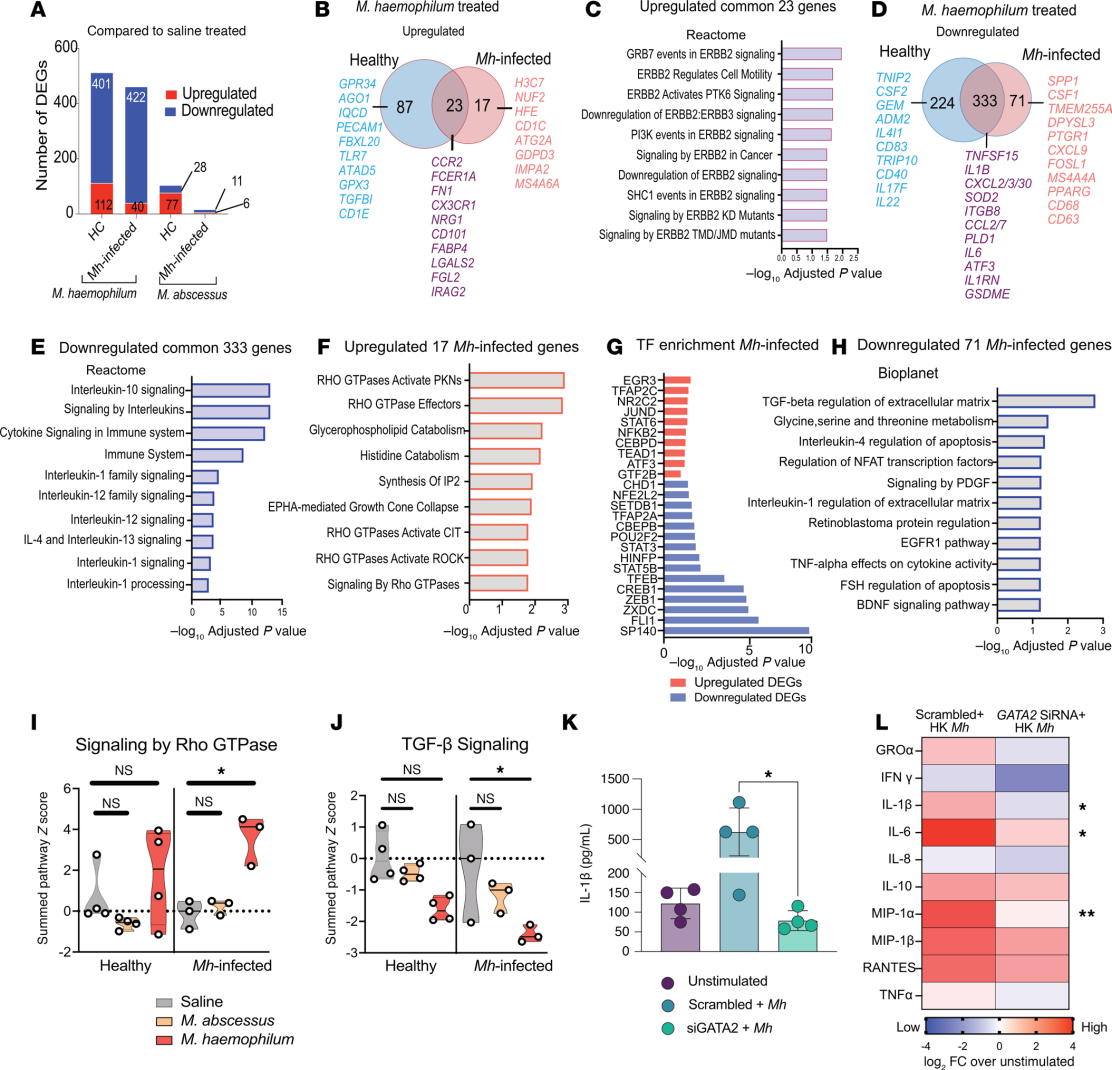
*Mh*-infected patients show impaired transcriptional response to *Mh* in vitro, which is GATA2 dependent. (**A**) Number of differentially expressed genes (DEGs) in response whole blood stimulation to HK *Mh* add *Mab* in HCs (*n* = 3) and *Mh*-infected patients (*n* = 4). (**B**) Overlap of upregulated genes to *Mh* stimulation in HC and MH-infected patients. (**C**) Reactome pathway enrichment of the common 23 genes in **B**. (**D**) Overlap of downregulated genes to *Mh* stimulation in HC and *Mh*-infected patients. (**E**) Reactome pathway enrichment of the common 333 genes in **D**. (**F**) The pathway enrichment of the 17 exclusively upregulated genes in *Mh*-infected (Reactome). (**G**) Pathway enrichment of the 71 exclusively downregulated genes in *Mh*-infected patients implemented in Enrichr (113, 115, 116). (**H**) Putative TF enrichment of up and downregulated genes exclusively regulated in *Mh*-infected individuals. (**I** and **J**) The summed *z*-pathway score of the top up- and downregulated pathway for each condition. Each dot represents an individual. Two-way ANOVA with Sidak’s multiple correction was used. *P < 0.05. (**K**) Reduced cytokine levels after *GATA2* knockdown using siRNA in human PBMCs (*n* = 4) as compared with scrambled and unstimulated controls. Kruskal-Wallis test with multiple comparisons is reported. Data are shown as mean ± SD. (**L**) Multiplex cytokine assay shows widespread reduction in inflammatory responses in healthy PBMCs (*n* = 2) upon GATA2 siRNA treatment to *Mh* stimulation. A heatmap of log_2_FC of cytokine and chemokine levels over the levels in unstimulated, showing scrambled versus siRNA treated PBMCs upon *Mh* stimulation. Two-way ANOVA results are shown with Fisher’s LSD. *P < 0.05, **P < 0.01.

To understand the lack of *Mh*-specific proinflammatory cytokine production upon reexposure in *Mh*-infected patients but retention of the proinflammatory response to nonspecific PHA and to other mycobacteria (*Mab*), we performed RNA-Seq analysis. Whole blood, obtained from 4 HC and 4 *Mh*-infected patients after treatment with either HK *Mh*, *Mab*, or saline for 24 hours, was analyzed for transcription differences (Supplemental Table 1 and Supplemental Figure 4). *Mh* treatment induced distinct transcriptional responses from saline and *Mab* treatment according to principal component analysis (PCA), with clear separation on the first component accounting for 40% of variance (Supplemental Figure 1A). The HC and *Mh*-infected patient baseline (saline treatment) transcriptional profiles were very similar in the PCA, and treatment with *Mab* induced similar transcriptional responses between HC and *Mh*-infected individuals (Supplemental Figure 1A). Transcriptionally, as compared with their respective untreated controls, stimulation of whole blood with HK *Mh* resulted in a high number of differentially expressed genes (DEGs; FDR-adjusted *P* ≤ 0.05) in both HC (112 up, 571 down) and *Mh*-infected individuals (40 up, 422 down). Conversely, the stimulation with *Mab* resulted in fewer DEGs in both HC (77 up, 28 down) and *Mh*-infected (6 up, 11 down) (Figure 3A and Supplemental Figure 2). At baseline, comparing HC to *Mh*-infected individuals with only saline treatment, there was only 1 upregulated DEG with *Mh* infection (Supplemental Table 6). The upregulated DEG was major histocompatibility complex class II DQ alpha 1 (*HLA-DQA1*, *P* = 0.0205). Polymorphisms in *HLA-DQA1* have been associated with protection and susceptibility to pulmonary TB in many studies (43). Two other downregulated DEGs were just above the FDR-corrected significance cutoff applied for all of the other comparisons (*P* = 0.0578 each) were thrombospondin 1 (*THBS1*, 15.5-fold down), which is known to protect against pathogen-induced lung injury by limiting extracellular matrix proteolysis (44) and *CD101* (5.086-fold down) an inhibitor of T cell proliferation induced by CD3. Interestingly, *CD101* was significantly upregulated with *Mh* treatment but not with *Mab* treatment in both the HC and MH-infected individuals (Supplemental Table 6).

The *Mab* treatment induced greater transcriptional changes in HCs with 105 DEGs (77 up, 28 down), while *Mh*-infected patients showed less changes in their transcriptional profile to *Mab* treatment showing a limited number of 16 DEGs (5 up and 11 down) (Figure 3A). Among the 75 genes significantly upregulated upon *Mab* treatment in HC (Supplemental Table 7) were genes including *VPS9D1*, a regulator of clathrin-mediated endosomes (45); ILs (*IL6/21/23*); *NR4A3*, a nuclear receptor whose expression is downregulated by *M*. *tuberculosis* (46); chemokines *CXCL1/2/3*; transcription factors such as *STAT5A*, *NFKB1/2*; IFN-regulated *GBP4* and genes related to energy metabolism; and the ROS balance-related enzyme gene *GOT1* (Supplemental Figure 1B). These 75 upregulated genes were enriched for pathways including TNF-α signaling; inflammatory response; cytokine signaling and IL responses, including IL-10 signaling (Supplemental Figure 1D and Supplemental Table 8); and the enrichment of transcription factors such as ZNF207, MYB, PPARG, NFE2L2, STAT3, and NF-κB1 (Supplemental Figure 1E and Supplemental Table 9).

Only 4 genes were upregulated after *Mab* treatment but not *Mh* in *Mh*-infected individuals, all of which were shared with HC (Supplemental Figure 1C and Supplemental Table 10). Fewer genes were downregulated upon *Mab* treatment (but not *Mh* treatment) in both HC (17 genes) and *Mh*-infected individuals (2 genes) (Supplemental Figure 1, B and C). For HC, the 17 downregulated genes included *LIPA*, *CYB1B1*, *RAB42*, *LGALS2*, *IDH1*, and B cell activation molecule *C180* and *CD36*, a class B scavenger receptor found in a wide variety of innate and adaptive immune cells (Supplemental Figure 1, B and C). These 17 genes were enriched for neutrophil degranulation, TLR4 cascade, and innate immune system pathways (Supplemental Figure 1D and Supplemental Table 10) as well as TFs including GATA1, GATA6, and IRF1 (Supplemental Figure 1E and Supplemental Table 12). The *Mh*-infected individuals showed significant downregulation of only 2 genes (*LIPA* and *CYB1B1*), which were also among the 17 downregulated in the HC.

A summed pathway *z* score of the top upregulated pathway, TNF-α signaling, showed that this pathway was upregulated only upon *Mab* treatment and remains downregulated upon *Mh* treatment (Supplemental Figure 1F). Similarly, the downregulated pathway of innate immune regulation showed *Mab*-specific downregulation (Supplemental Figure 1G). The DEGs between *Mh-* and *Mab*-treated whole blood showed minimum overlap in either HC or *Mh*-infected individuals (Supplemental Figure 1, B and C), indicating that, in general, the human whole-blood transcriptional response to *Mh* and *Mab* is different (Supplemental Figure 1, A–C). This was also apparent in the opposite regulation of the top genes between the 2 treatments, and 23 such DEGs were identified that showed upregulation upon *Mab* treatment and downregulation with *Mh* treatment in both HC and *Mh*-infected patients (Supplemental Figure 1H). These included inflammatory cytokine and chemokine genes such as *CXCL1/2/3/5*, *IL6*, *IL1B*, *CCL20*, *IL21*, *IRAK1*, *IL1A*, *SOCS3*, *MMP14*, and *NFKB1/2* (Supplemental Figure 1H). These genes were enriched in IL-10 signaling, IL-1 signaling, NF-κB activation and inflammasome pathways. These pathways, therefore, would be up regulated upon *Mab* and downregulated upon *Mh* treatment (Supplemental Figure 1I). The upregulation of the IL-10 signaling and genes such as *SOCS3* also indicate that the inflammatory signaling to *Mab* is being counterbalanced by antiinflammatory responses.

Next, we identified gene sets that were significantly differentially regulated by *Mh* treatment but not *Mab* treatment (Supplemental Figure 2, A and B). HC and *Mh*-infected individuals both showed a large overlap among these upregulated (23 genes) and downregulated (333 genes) gene sets (Figure 3, B–D). The top commonly upregulated genes included *CCR2*, which serves an early and essential role in resistance to *M*. *tuberculosis* (47); *FCER1*, the initiator of the allergic response (48); and *FN1*, which facilitates *M*. *tuberculosis* attachment to murine alveolar macrophages (49) (Supplemental Table 13). The commonly upregulated 23 genes showed Reactome enrichment of ERBB2 signaling pathways (Figure 3C and Supplemental Table 14), which are part of tyrosine kinase receptors and regulate host cell entry in *M*. *leprae* (50); they also prevent macrophage function, leading to enhanced infection with *M*. *tuberculosis* (51). The commonly downregulated 333 genes included cytokine- and chemokine-related genes including *TNFSF15*, *IL1B*, *CXCL2*, *CXCL3*, *CXCL30*, and *IL6* (Figure 3D and Supplemental Table 15), which then were also enriched in IL-related pathways including IL-10, IL-1, IL-12, IL-13 signaling (Figure 3E and Supplemental Table 16). Interestingly, IL-6 is upregulated upon *Mab* response but downregulated with *Mh* treatment (Supplemental Figure 1, B, C, and G). Similarly, enrichment of IL-10 signaling, which can suppress antimycobacterial immunity and promote the survival of pathogen (52) (Figure 3E), was upregulated upon *Mab* treatment and downregulated with *Mh* treatment. Collectively, both HCs and *Mh*-infected patients show common downregulation of cytokine and chemokine signaling to *Mh* stimulation.

Among the genes differential in only HC (and not *Mh* infected) upon *Mh* stimulation (and not *Mab*), 87 genes were upregulated and 224 genes were downregulated (Figure 3, B and D). Of the 87 genes that were upregulated only in HC, the following were included: *GPR34*, a regulator of macrophages activation and phagocytic activity, and *TLR7*, which recognizes single-stranded RNA and *TGFBI*, associated with TGF-β signaling (Figure 3B and Supplemental Table 17). Interestingly, these genes were enriched in cholesterol transport and efflux (Supplemental Table 18). Among the 224 HC specific downregulated genes, upon *Mh* stimulation were genes including *TNIP2*, a regulator of NF-κB signaling (53); *CSF2*, also known as GM-CSF an important regulator of differentiation and cytokine production in granulocytes and macrophages (54); cytokine genes such as *IL17F*, *IL22*, and *IL4I1*; and *CD83*, which is an important immune checkpoint molecule (55), critical for resolution of inflammation (56) (Figure 3D and Supplemental Table 19). These downregulated genes enriched in cytokine, IL signaling, and TNF receptor and neutrophil degranulation (Supplemental Table 20).

Similarly, we identified 17 upregulated and 71 downregulated DEGs in *Mh*-infected individuals (but not HC) by *Mh* treatment but not *Mab* treatment (Figure 3, B and D, and Supplemental Tables 21 and 22). These 17 upregulated genes included gene such as *H3C7*, *NUF2*, *HFE*, *CD1C*, autophagy related *ATG2A*, *GDPD3*, and the M2-macrophage related gene *MS4A6A* and were enriched primarily in the regulation of RHO GTPases pathway (Figure 3F) and enriched in TF regulators including JUND, STAT6, NF-κB2, and ATF3 (Figure 3G). Among the 71 downregulated genes only in *Mh*-infected patients, the following were included: *SPP1*, or osteopontin, a gene associated with function of perivascular macrophages (57); *CSF1* (also known as M-CSF), a factor that controls cytokine production and macrophage survival (54); chemokine such as CXCL9; *MS4A4A*, expressed in tissue resident macrophages (58); and monocytic lineage marker *CD68* and *CD63*, an endosomal marker downregulation of which is associated with mycobacterial persistence (59) Employing 4 additional pathway databases for enrichment of the 71 downregulated genes revealed Mh-individual specific enriched pathways. Interestingly, among the 4-database search included, which were applied using Enrichr, downregulation of TGF-β (Panther and Bioplanet databases) and TNF-α signaling (Bioplanet and Hallmark databases) in *Mh*-infected patients emerged as a common theme (Figure 3H and Supplemental Table 23). Linking this to the LOF mutation in the *GATA2* gene (Figure 2D), it is known that TGF-β is an important regulator of inflammation and is negatively regulated by GATA2 (60). This TGF-β–GATA2 association is important for human NK cell development (61), and a lower number of NK cells associates with TB disease (62). Other pathways enriched among these 71 downregulated genes included PI3K/mTORC1 signaling and glycine metabolism. Interestingly, among the KEGG pathways, there was downregulation of C-type lectin signaling (Supplemental Table 23), which are important sensors of mycobacterial infections, binding to multiple ligands, downregulation of which could be an important to *Mh* responses as well. The downregulated pathways enriched for TF included ZEB1, CREB1, STAT5B, STAT3, NFE2L2, and SP140 (Figure 3H). Interestingly, SP140 which is an important negative regulator of Type I IFN and an essential transcriptional regulator for resistance to bacterial infections (63), was one of the most significant predicted TF for the downregulated genes. Therefore, the downregulated genes upon *Mh* responses in *Mh*-infected individuals might be inducing a super susceptible phenotype in these persons and could be tied to a dysregulated Type I IFN response. A summed pathway score of the most significant pathways — Rho GTPase for upregulated (Figure 3I) and TGF-β signaling for downregulated genes (Figure 3J) — shows that, indeed, these pathways are up- and downregulated, respectively, and that this is specific to *Mh* treatment and not *Mab* treatment (Figure 3, I and J). An interesting observation was the enrichment of TLR4 signaling (downregulation of accessory molecules *CD180* and *CD36*; refs. 59, 64) for downregulated genes upon *Mab* stimulation (Supplemental Figure 1D) among HC, while there was an upregulation of *TLR7* in HC upon *Mh* stimulation (Figure 3B). TLR signaling is important for inflammatory signaling against pathogenic mycobacteria (65). In line with that, *M*. *avium* infection of human primary macrophages signals through TLR7/8 and is dependent on MyD88, TF such as NF-κB and IRF1 (59). While HC seem to induce high expression for *TLR7* (2.7-fold; *P* = 9.4 × 10^–5^), the *Mh*-infected patients fail to upregulate *TLR7* significantly (0.79-fold, nonsignificant), upon *Mh* stimulation, while *Mab* stimulation does not significantly change *TLR7* expression in HC or *Mh*-infected patients (Supplemental Figure 3A). Interestingly, GATA2 deficiency in fetal progenitor cells is linked to elevated levels of TLR transcripts (66). In our results, in *Mh*-infected patients with GATA2 deficiency, only *TLR7* showed baseline slight upregulation (1.4 FPKM in HC versus 1.8 FPKM in *Mh*-infected patients), but this was almost similar to *TLR 2*, *4* and *8* transcript levels (Supplemental Figure 3, B–D).

To address if the GATA2 deficiency was a driver of the attenuated cytokine phenotype from HK *Mh*, we used siRNA knockdown of *GATA2* in peripheral blood cells (PBMCs) from independent healthy controls. We observed a significant reduction in the levels of IL-1β, IL-6, and MIP1a (*P* < 0.05) in *GATA2* siRNA–treated PBMCs as compared with scrambled controls, which showed induction of these cytokines upon HK *Mh* stimulation (Figure 3, K and L).

## Discussion

The pathophysiology of *Mh* infection is not very well understood. Extensive disease from *Mh* is rare in healthy individuals and usually seen in immunocompromised individuals (bone marrow transplant, HIV infection, malignancies) (12) and rarely in individuals with mutations in regulatory genes (67). *Mh* infection in immunocompromised patients commonly manifests as cutaneous lesions, pyomyositis, disseminated pulmonary infection, ophthalmologic manifestation, and osteomyelitis (10), and CNS infection has been reported on isolated cases for *Mh* in patients with AIDS (18) and renal transplant (19). Here we report the characterization of genomic, transcriptional, and cytokine responses from *Mh*-infected patients (3 confirmed, 1 suspected) with CNS and cutaneous manifestation from the same geographical area (Missouri, USA). The patients had weakened (Patient 1) (22) or immunocompromised state (Patients 2, 3, 4); they showed vulnerability to *Mh* with infections in their CNS and skin and the results highlight mutations in the *GATA2* gene (p.A164T, rs2335052), as the possible driver of a *Mh* susceptible phenotype in these individuals. Whole blood of the susceptible individuals had a severely attenuated proinflammatory response, typified by lower levels of cytokines and chemokines (IL-8, TNF-α, IL-1β, IL-6, MIP1-a, CXCL1, and G-CSF) to in vitro stimulation to HK *Mh*, but not PHA or *Mab*. Transcriptional profiling confirmed our observation of a *Mh*-specific defect in these individuals, with minimal overlap to transcriptional response to *Mab*. In summary, we present exome, functional cytokine, and transcriptome-level data to decipher the susceptible phenotype in *Mh*-infected individuals and identify specific defects in immune signaling as the reason for the susceptibility. Additionally, we present a comparative transcriptome analysis between *Mh* and *Mab* and describe differential transcriptional responses in both healthy and *Mh*-infected individuals. Finally, we validated that siRNA knockdown of *GATA2* in healthy PBMCs attenuated cytokine response to HK *Mh*, similar to *Mh*-infected individuals, showing loss of *GATA2* resulted in attenuated cytokine profiles.

*Mh*, a slow-growing acid-fast bacillus, is different from other mycobacterial species, prefers a lower growth temperature, and requires iron supplements (16, 17). The diagnosis is challenging and, based on index of suspicion when in a suspected case the AFB^+^ smear, fails to grow under normal culture conditions. No specific antigen test is available for *Mh* infections. Direct detection of *Mh* in clinical materials is employed through either a PCR with subsequent restriction analysis of *hsp65* gene (68) or using real-time PCR (69). The PCR results should be corroborated with clinical presentations, due to widespread presence of NTM in the environment, and can result in false positives. The 4 presented cases in the study underwent the described diagnosis starting with a high index of suspicion, followed by confirmations via PCR, reiterating the challenges faced during NTM confirmation and diagnosis (10, 22).

Examining host genomes and immune responses has led to identification of 16 critical genes majorly leading to IFN-γ defects (14) in a congenital condition called Mendelian susceptibility to mycobacterial disease (MSMD). While MSMD is usually associated with NTM such as *Mab*, *M*. *bovis*, *M*. *elephantis*, *M*. *fortuitum*, *M*. *simiae*, and *M*. *chelonei* (13), such defects for *Mh* are not reported. In this study, we show that *Mh*-infected individuals exhibit enrichment of specific nonsynonymous mutations, which were lacking in HCs. Interestingly, iron is required for *Mh* growth in culture, so the *SLCO2B1* could be an important susceptibility gene for *Mh* exposure. Exploring the REVEL (70) pathogenicity scores, all 4 *Mh*-infected individuals harbored the same nonsynonymous mutations in the *GATA2* gene, showing the highest score, followed by *GPR35* and *GGH* gene. *GATA2* is an important transcription factor and a critical regulator of hematopoietic stem cells emergence and proliferation (71). Mutations in this gene have been related to clinical syndromes such as myelodysplastic syndrome and acute myeloid leukemia, including increased susceptibility to NTM (72) such as *M*. *kansasii* (73), *M*. *avium* (74)—complexes typified by as MonoMAC syndrome (39, 67). While *GATA2* mutations have been linked to other NTMs such as *M*. *kansasii*, *Mab*, *M*. *simiae* and *M*. *avium*, *M*. *chelonae*, *and M*. *genavense* (75, 76), there have been no prior reports linking it to *Mh*. Interestingly, these *Mh*-infected individuals had none of the known MSMD mutations. The specific mutation in *GATA2* identified in all 4 *Mh*-infected cases in the coding third exon (p.A164T, rs2335052) has been linked to *M*. *kansasii* and *M*. *fortuitum* and infection recently reported in patients with *Mh* infection, but it was classified as “benign” (nondisease causing) (77). In contrast, the same mutation found in all 4 *Mh*-infected patients in our study associates with an immune-response–deficient phenotype. So here we describe *Mh* infections in individuals with *GATA2* mutations (p.A164T, rs2335052) for the first time to our knowledge.

Cytokine responses to weakly virulent environmental mycobacteria can be elicited by distinct or shared pathway activation with virulent mycobacteria. For example, cytokine responses to *M*. *avium* is linked to MyD88-dependent TLR2/4 signaling (shared with *M*. *tuberculosis*) (78), the magnitude of which is higher for *Mab*, which leads to the production of proinflammatory cytokines such as TNF-α, IFN-γ, IL-6, MIP-1α, and RANTES. *Mab* responses, as such, are driven by ERK1/2 signaling and NF-κB translocation (79). As expected, *Mab* stimulation in this study induced elevated levels of proinflammatory cytokines and chemokines in both HCs and *Mh*-infected individuals, indicating that the immune response defect is distinct and specific to prior *Mh* exposure. On the contrary, *Mh*-infected individuals showed attenuated cytokine responses to *Mh* stimulation compared with the HC responses. The differences in response to *Mh* stimulation can be due to differences in the antigen composition. For instance, *Mh* has been shown to have phenolic glycolipid (PGL) antigen, with similar lipid core *M*. *leprae*, *M*. *kansasii*, and *M*. *tuberculosis* but unique trisaccharide moiety not found in other mycobacteria (80). The oligosaccharide renders the antigen uniqueness, as such PGL from *M*. *bovis*, *M*. *canetti*, and *M*. *leprae* induce different magnitude of iNOS production in BMDMs and act by disrupting the TLR4 signaling (81). Similarly, we observe attenuated *TLR7* levels in the *Mh*-infected individual’s transcriptome, which could mean that the sensing of *Mab* is known to be TLR2/4 driven and that *TLR7* seems to be guiding *Mh* stimulation signaling. Linking the attenuated cytokine response to mutation in *GATA2*, it is known that GATA transcription factors can inhibit cytokine levels by binding to STAT3 (82), which coincides with enrichment of STAT3 among downregulated genes in *Mh*-infected individuals. Similarly, *Gata2*-heterozygous mutant mice also showed attenuated inflammatory cytokine responses, with LPS stimulation and decreased bacterial clearance (83). Such observation lends credibility to our argument that *GATA2* mutation is possibly driving the attenuated cytokine phenotype in *Mh*-infected individuals.

Transcriptionally, we identified, a large overlap between the HCs and *Mh*-infected patients to *Mh* stimulation. This indicates that these responses are not affected by the presence of LOF and/or pathogenic mutations identified in *Mh*-infected individuals. This also makes them core responses in whole blood induced by *Mh* infection and provides us insight into *Mh* host responses. The commonly upregulated genes enriched predominantly into ERBB2 signaling. Interestingly, ERBB2 receptor has roles in mycobacterial infections, with *M*. *leprae* directly binding ErbB2 to enter the host cells (50), while *M*. *tuberculosis* induced ErbB2, preventing proper macrophage function and increased infection (51). *M*. *leprae* is phylogenetically (10) close to *Mh*, so that could explain similar host mechanism enrichment observed fin *Mh*-infected individuals. *Mh* stimulation resulted in widespread downregulation of the IL signaling pathways, in both HCs and *Mh*-infected individuals, which was reflected in lower serum levels of the cytokines. While the *IL1B* transcripts were downregulated in both groups upon *Mh* stimulation, serum IL-1β levels remained high in HC but lower in *Mh*-infected individuals, indicating that the immune downregulation, though shared, is more pronounced for *Mh*-infected as compared with HC.

The transcriptional responses to Mab differed from *Mh*, with more genes upregulated than downregulated — particularly in TNF signaling, cytokine signaling, inflammatory responses, and IL responses. This pattern resembles the cytokine-mediated induction reported during Mab infection of THP-1 macrophages, which led to elevated expression of TNF-α, CCL4, IL-8, and IL-1β (84). *Mh*-infected individuals showed minimal transcriptional change as compared with HC but had a similar elevated cytokine profile to *Mab* stimulation. Notably, 23 inflammatory genes were inversely regulated, with *Mab* inducing upregulation, and *Mh* caused downregulation in both HCs and *Mh*-infected patients. These genes may serve as transcriptional biomarkers to distinguish infection by these pathogens. *Mh-* and *Mab*-stimulated genes showed enrichment of specific transcription factors, further indicating that their transcriptional signature is different. Among the predicted TF for *Mh*-upregulated genes were JUND, STAT6, and NF-κB2, while for *Mab*, they were PPARG, NFE2L2, STAT3. Interestingly, NFE2L2 is enriched among upregulated genes upon *Mab* stimulation but not among downregulated genes upon *Mh* stimulation. JUND (activator protein-1 family) regulates IL-1β (85), IL-6, MMP-1 (86), TNFA (87), and COX2 (88) all of which are critical for inflammation and immune responses, including during mycobacterial infections (89–92). STAT6, activated by IL-4 and IL-13, regulates IL-4, IL-5, IL-13, and CCL17, which are central to Th2-mediated responses in allergic inflammation (93). Interestingly STAT6 inhibits Mincle expression and polarizes towards a Th2 environment (94). NF-κB2 is a central TF activated by multiple stimuli and regulates TNF-α, IL-1β, IL-6, CXCL16, MMP-9, and ICAM-1, which play pivotal roles in immune activation, inflammation, and recruitment of immune cells (95). NF-κB is critical to mycobacterial control (96, 97).

The transcription factors enriched for *Mab* upregulated genes were PPARG, NFE2L2, and STAT3. PPARG modulates IL-10 (98), TNF-α (99), and NOS2 (100), promoting antiinflammatory responses and macrophage polarization toward an M2 phenotype (101). PPARG via 15-LOX regulate macrophage apoptosis during *M. tuberculosis* infection (102). NFE2L2 (NRF2) is a master regulator of host antioxidant response and interacts with KEAP1, HO-1 (HMOX1), GCLM, and BACH1, as well as NQO1 for its effector function (103). NRF2 activation has been linked to protective immunity against *M. tuberculosis* (104). STAT3 signalling regulates multiple target genes including cytokines CCL5, CXCL10, IL-6/10, TNF, IFN-γ, IL12, and genes such as AKT, MMP2, MMP9, and Vimentin (105) to name a few, showing roles in cell proliferation, survival, and differentiation. STAT3 interacts with IL-6 and NF-κB to drive inflammatory responses (105). STAT3 with its downstream gene SOCS3 are major players in shaping T cell responses and control of *M. tuberculosis* (106).

Considering transcriptional responses exclusive to *Mh*-infected individuals and linking them to mutations in *GATA2*, we observe downregulation of genes including *CSF1*, the colony stimulating factor 1 or Macrophage colony stimulating factor (M-CSF). This factor plays a critical role in GATA2, including myeloid cell differentiation and as a transactivation of GATA2 responsive promoters, to induce transcription of effectors. *CSF1* downregulation in *Mh*-infected people harboring loss-of-function GATA2 mutation upon *Mh* restimulation is interesting, as mutant *GATA2* fails to transactivate GATA2 responsive promotors such as *CSF1R* in humans (107, 108). Although not the same mutation, failure of mutant *GATA2* to transactivate CSF1R could explain lower *CSF1* transcripts and could correlate to lowered innate immune responses and cytokine levels, as observed in this study.

As discussed earlier, *Mab* is sensed through TLR2/4 pathway (109), which was downregulated upon *Mab* stimulation. Similarly, from our transcriptional data, it appears that TLR7 might be used for sensing of *Mh*, as *Mh* but not *Mab* treatment significantly upregulated *TLR7* in HC. *Mh*-infected individuals showing significant downregulation of *TLR7* and *TLR8* transcripts and not *TLR2* and *TLR4* transcripts, showing that *Mh*-infected individuals that harbor GATA2 mutations have downregulation of *TLR7/8* signaling. This fits with the paradigm of absence of GATA2 regulating the TLR signaling and sensing (66). Our findings therefore suggest that fundamental differences in the mycobacterial sensing and downstream signaling might be driving the transcriptionally observed differences in *Mab* and *Mh* stimulations. On the immune response front, the response to *Mh* is likely driven by a functional *GATA2* gene, as knockdown of *GATA2* resulted in attenuated cytokine responses.

## Methods

### Sex as a biological variable.

Our study looked at rare occurrence of the *Mh* infection in 4 individuals and all were Male. Owing to the rarity of the disease phenotype, sex had no role in study design and not considered as a biological variable.

### Study design.

In total, 4 confirmed infected (*Mh*-infected) and 4 HC donors were recruited for the study (Table 1). The aim of this study was to develop a better understanding of the patients’ immune responses to *Mh* infection and investigate possible common genetic basis for these uncommon infections. To accomplish this, we incorporated a whole blood stimulation assay as an in-vitro model, whole exome sequencing, and RNA sequencing which are described below. Since this was a study on determining the genetic etymology of a disease which is not very well understood, a sample size determination was not possible, and all incoming patient with similar disease presentation and later confirmation for *Mh* infection were included.

### Blood collection and deidentification.

Consented donors were deidentified at the clinic. Approximately 10 mL of venous blood was drawn into sodium-heparin tube (green top) and rapidly inverted 7 or 8 times to mix, at the time of donor recruitment. The blood was brought to research lab with 2 hours of withdrawal, for downstream processing. Downstream processing was started immediately.

### Bacterial culture and heat inactivation.

*Mh* was procured from American type culture collection (ATCC) (Cat # 29548) and was grown as per instruction. Briefly, lyophilized bacteria were reconstituted and cultured in Middlebrook 7H9 broth supplemented with albumin-dextrose catalase (ADC; 10% v/v) and hemin (0.039 gm/L) at 30°C incubator with constant agitation. Late log phase culture was harvested, aliquoted and cryopreserved. *Mab* was cultured similarly (without hemin) at 37°C. The colony forming units (CFU) was estimated by dilution plating on Middlebrook 7H10 agar plates. For heat-inactivation, bacterial stocks were thawed, washed, and resuspended in Phosphate Buffered Saline. Aliquots of 200 mL were boiled at 95°C for 20 minutes. Total protein was estimated by BCA protein estimation kit (Pierce; Cat # 23250). *Mab* culture was brought to 1.2 × 10^6^ CFU/mL prior to heat-inactivation.

### Ex-situ whole blood stimulation assay.

Whole blood (450 mL) from HC as well as *Mh*-infected individuals were transferred to 2mL tubes (Sarstedt Micro Tube Silicone; Sterile). Within 2 hours of blood withdrawal, costimulatory antibodies CD49d and CD28 (Becton and Dickinson Cat # 340976 [clone: L25] and 340975 [clone: L293]) and heat inactivated *Mh* (prepared as described earlier) was added at 3 different concentrations: 50 mg/mL, 75 mg/mL and 100 mg/mL. Phytohemagglutinin (PHA; 10 mg) and *Mab* (100 mg/mL) served as controls in the assay setup; negative control (NC) for the experiment was adding the costimulatory antibodies (CD49b and CD28) alone in absence of any stimulation. Additionally, Saline was added as a control. Following 12 hours of incubation with respective stimulants, plasma was collected and stored at –20°C for cytokine analysis.

### Cytokine analysis.

Cytokine/chemokine secreted in human plasma post invitro assay was analyzed via MILLIPLEX Multiplex Assay kit (MilliporeSigma) as per the manufacturer’s protocol and the assay plate was read using Bio-plex Luminex (Bio-Rad).

### Whole blood DNA isolation.

DNA was prepared immediately upon arrival of whole blood from HCs and infected donors, and subjected to the DNA isolation according to manufacturer protocol (Qiagen DNA isolation kit; Cat # 69504), quantitated using Nanodrop microvolume spectrophotometer (ND-100, Thermo Fisher Scientific) and submitted for whole exome sequencing (WES) at the genomics core of the McDonnell Genome Institute, Washington University in St. Louis, St. Louis, MO, USA.

### Exome sequencing and data processing.

Germline samples from 4 *Mh*-infected individuals and 4 HCs were sequenced to an estimated coverage of 50× and aligned using bwa (110) against human reference genome (GRCh38/hg38). The sequencing data was processed as described in supplementary detailed methods.

### RNA-Seq of ex situ stimulated donors blood cells.

Cells from whole blood stimulation assay were cryopreserved which were thawed and subjected to RNA preparation and bulk sequencing.

### RNA-Seq processing and analysis.

RNA was prepared and the sequenced Fastq files were processed as previously described (111, 112) (Supplemental Methods). Processed sequencing data are provided in Supplemental Table 1. Pathway enrichment analysis was performed as previously described (111). Additionally, Enrichr was used for pathway and transcription factor analysis (113). All considered pathways and TF were significant (*P* < 0.05).

### In vitro GATA2 knockdown.

PBMCs from independent healthy donors were left untreated, treated with Scrambled (Thermo Fisher Scientific, Cat # 4390843) or human GATA2 siRNA (Thermo Fisher Scientific, Cat # 4392420). The PBMCs (n =4) were transfected using the HiPerFect transfection Reagent (Qiagen, Cat # 301704). The duration of treatment and the concentration of siRNA were titrated to attain at least 50% knockdown of the GATA2 protein which was confirmed by Flow-cytometry staining of GATA2. The cells treated with optimized concentration of 200 nM of the siRNA for 24 hours; then, the reaction was stopped by adding complete media (10% human AB serum) and cells allowed to recover overnight. The next day cells were stimulated with HK *Mh* (100 mg), the highest concentration used for the whole blood assay for 24 hours. The supernatant collected and evaluated by ELISA for IL1b (R&D systems) (*n* =4). The optical density obtained at 450nM was extrapolated from a standard curve to get the cytokine levels (pg/mL). To elucidate the magnitude of the effect of siRNA knockdown multiplex ELISA was run on (*n* = 2) using the MILLIPLEX Human cytokine/chemokine/Growth factor Panel A (Cat # HCYTA-60K) following manufacturer’s instruction. The multiplex was acquired on Luminex 200 Instrument.

### Statistics.

The appropriate statistical analysis is explained in the sections as they appear for Exome sequencing and RNA-Seq. There was no blinding needed for the analysis. For analysis of the cytokine data, 1-way ANOVA was applied to compare among the groups for with Tukey’s test for multiple testing correction. For pairwise analysis, a 2-tailed unpaired *t* test was used. Two-tailed *P* < 0.05 was considered significant. Summed *z*-scores were calculated for pathways using the median log_2_ fold change of all the enriched genes in the pathway per individual using their respective saline control. Summed *z*-score differences were tested using Two-way ANOVA with sidak’s multiple correction test. *P* < 0.05 was considered significant.

### Study approval.

The study was conducted over a 4-year period (2017–2021) at the Washington University in St. Louis, School of Medicine, and approved by IRB approval no. 201811050. The validation study was approved by the IRB at UChicago, approval no. 230069. Informed written consent was obtained from all the participants.

### Data availability.

The sequencing data has been deposited to the European genome phenome Archive (EGA) and can be accessed upon request from the EGA. The accession number for the Exome data is EGAS50000001076, and the RNA-Seq data is EGAS50000001077. All other supporting data are supplied Supplemental tables and the Supporting Data Values.

## Author contributions

Conceptualization, overall supervision, data visualization and validation, and manuscript preparation were contributed by SAK. Protocol standardization, experimentation, data generation, processing, and manuscript preparation were contributed by AG, A, ST, MA, MM, BAR, FF, GKA, and JM. Clinical coordination, experimentation, and manuscript preparation were contributed by TCB, DBC, and SBM. All authors reviewed and approved the manuscript. AG, SBM, and A contributed equally to this work. AG conducted initial in vitro experiments and RNA-Seq sample preparation. SBM led patient enrollment, diagnosis, and clinical coordination. A performed additional analysis and generated the figures, helped with writing up of these results, formatted the paper, and completed additional experiments during revision. The co–first authorship designation was agreed upon by all coauthors.

## Supplementary Material

Supplemental data

Supplemental tables 1-21

Supporting data values

## Figures and Tables

**Table 1:**
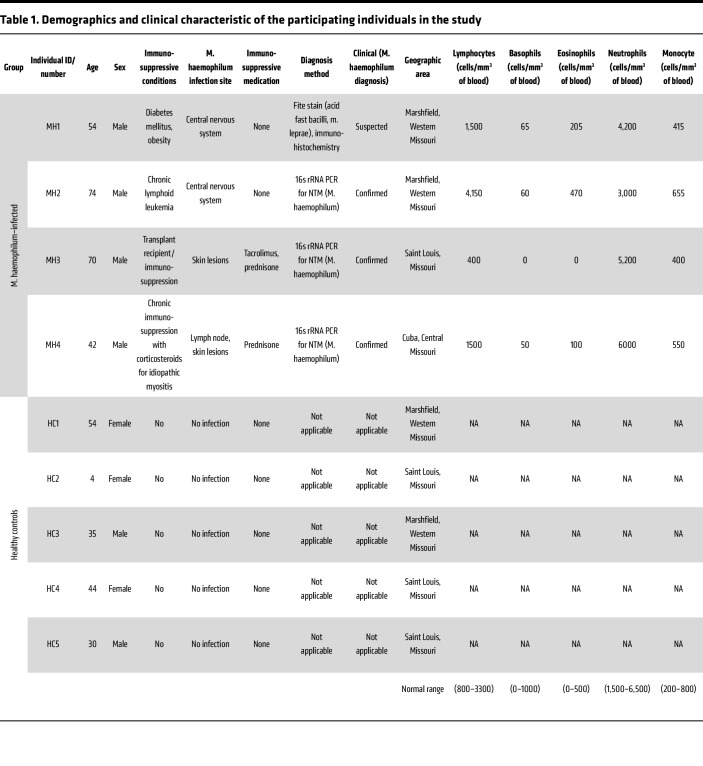
Demographics and clinical characteristic of the participating individuals in the study

## Version 1 08/05/2025

In-Press Preview

## Version 2 09/23/2025

Electronic publication
