# Supplementary material for: GATA2 mutation is associated with immune dysfunction and increased Mycobacterium haemophilum susceptibility in immunocompromised individuals
Source: JCI Insight. 2025 Aug 5;10(18):e185582. doi: 10.1172/jci.insight.185582 (PMC12487866; doi:10.1172/jci.insight.185582)
Supplement: Supplemental data [file jciinsight-10-185582-s268.pdf]

GATA2 mutation is associated with immune dysfunction and increased _Mycobacterium haemophilum_ susceptibility in immunocompromised individuals.

Authors: Ananya Gupta[#], Shail B. Mehta[#], Abhimanyu[#], Bruce A. Rosa, John Martin, Mushtaq Ahmed, Shyamala Thirunavukkarasu, Farheen Fatma, Gaya Amarasinghe, Mitreva Makedonka, Thomas Bailey, David B. Clifford, Shabaana A. Khader

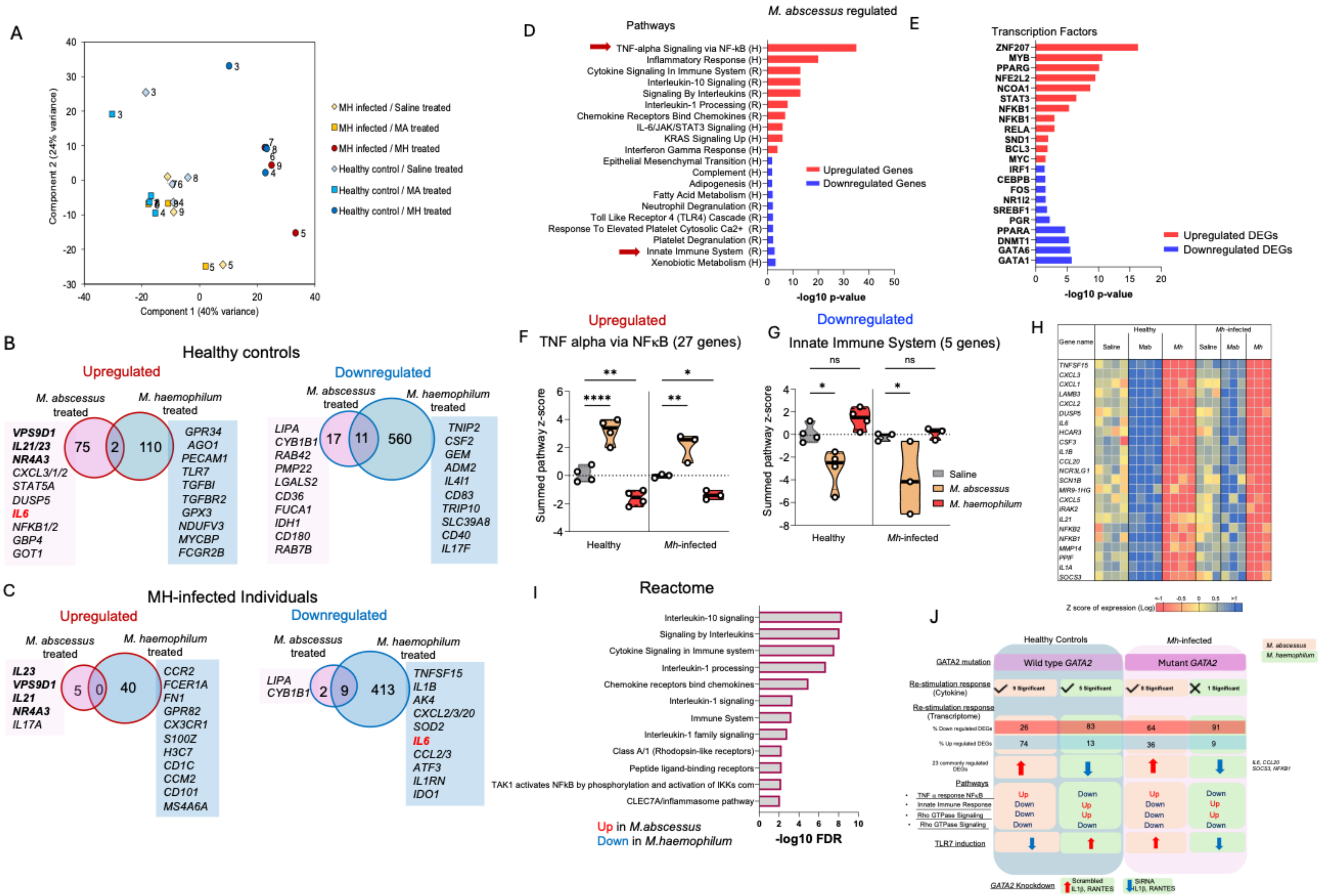

A
Component 2 (24% variance)
Component 1 (40% variance)
MH infected / Saline treated
MH infected / MA treated
MH infected / MH treated
Healthy control / Saline treated
Healthy control / MA treated
Healthy control / MH treated
B
Healthy controls
Upregulated
M. abscessus treated
M. haemophilum treated
75
2
110
VPS9D1
IL21/23
NR4A3
CXCL3/1/2
STAT5A
DUSP5
IL6
NFKB1/2
GBP4
GOT1
GPR34
AGO1
PECAM1
TLR7
TGFBI
TGFBR2
GPX3
NDUFV3
MYCBP
FCGR2B
Downregulated
17
11
560
LIPA
CYB1B1
RAB42
PMP22
LGALS2
CD36
FUCA1
IDH1
CD180
RAB7B
TNIP2
CSF2
GEM
ADM2
IL4I1
CD83
TRIP10
SLC39A8
CD40
IL17F
C
MH-infected Individuals
Upregulated
5
0
40
IL23
VPS9D1
IL21
NR4A3
IL17A
CCR2
FCER1A
FN1
GPR82
CX3CR1
S100Z
H3C7
CD1C
CCM2
CD101
MS4A6A
Downregulated
2
9
413
LIPA
CYB1B1
TNFSF15
IL1B
AK4
CXCL2/3/20
SOD2
IL6
CCL2/3
ATF3
IL1RN
IDO1
D
M. abscessus regulated
Pathways
TNF-alpha Signaling via NF-kB (H)
Inflammatory Response (H)
Cytokine Signaling In Immune System (R)
Interleukin-10 Signaling (R)
Signaling By Interleukins (R)
Interleukin-1 Processing (R)
Chemokine Receptors Bind Chemokines (R)
IL-6/JAK/STAT3 Signaling (H)
KRAS Signaling Up (H)
Interferon Gamma Response (H)
Epithelial Mesenchymal Transition (H)
Complement (H)
Adipogenesis (H)
Fatty Acid Metabolism (H)
Neutrophil Degranulation (R)
Toll Like Receptor 4 (TLR4) Cascade (R)
Response To Elevated Platelet Cytosolic Ca2+ (R)
Platelet Degranulation (R)
Innate Immune System (R)
Xenobiotic Metabolism (H)
Upregulated Genes
Downregulated Genes
-log10 p-value
E
Transcription Factors
ZNF207
MYB
PPARG
NFE2L2
NCOA1
STAT3
NFKB1
NFKB1
RELA
SND1
BCL3
MYC
IRF1
CEBPB
FOS
NR1I2
SREBF1
PGR
PPARA
DNMT1
GATA6
GATA1
Upregulated DEGs
Downregulated DEGs
-log10 p-value
F
Upregulated
TNF alpha via NFκB (27 genes)
Summed pathway z-score
Healthy
Mh-infected
G
Downregulated
Innate Immune System (5 genes)
Saline
M. abscessus
M. haemophilum
H
Gene name
Healthy
Mh-infected
Saline
Mab
Mh
Z score of expression (Log)
I
Reactome
Interleukin-10 signaling
Signaling by Interleukins
Cytokine Signaling in Immune system
Interleukin-1 processing
Chemokine receptors bind chemokines
Interleukin-1 signaling
Immune System
Interleukin-1 family signaling
Class A/1 (Rhodopsin-like receptors)
Peptide ligand-binding receptors
TAK1 activates NFkB by phosphorylation and activation of IKKs com
CLEC7A/inflammasome pathway
Up in M.abscessus
Down in M.haemophilum
-log10 FDR
J
Healthy Controls
Mh-infected
GATA2 mutation
Wild type GATA2
Mutant GATA2
Re-stimulation response (Cytokine)
Re-stimulation response (Transcriptome)
Pathways
TLR7 induction
GATA2 Knockdown

Figure S1

Fig.S1: Transcriptional response is distinct to *M. haemophilum* and *M. abscessus* stimulation in both HC and *Mh*-infected patients. A) Principal component analysis (PCA) expression of transcriptional profiles from Saline treated (green ellipse) , *M. abscessus* (yellow ellipse) *and M. haemophilum* (red ellipse) stimulation of whole blood in HC and MH-infected individuals; B) overlap of DEGs between *M. haemophilum* and *M. abscessus* as compared to respective saline treatment in HCs; and C) *Mh*-infected individuals; D) Top enriched pathways from up and downregulated genes from Hallmark (H) and Reactome (R). Negative log10 p-value is reported of $p < 0.05$, E) Top predicted transcription factors (TF) regulating both up and downregulated genes; F) The summed z-pathway score of the top up and downregulated pathway is shown. Each dot represents an individual. Two-way ANOVA with Sidak's multiple correction was used and $p<0.05$ is reported. *$p < 0.05$. G) Heatmap of 23 genes commonly regulated genes, showing distinct transcriptional profiles with *M. haemophilum* and *M. abscessus* treatment; H) Enriched Reactome pathways for the 23 genes in (G); J) A summary of major findings and themes of the study.

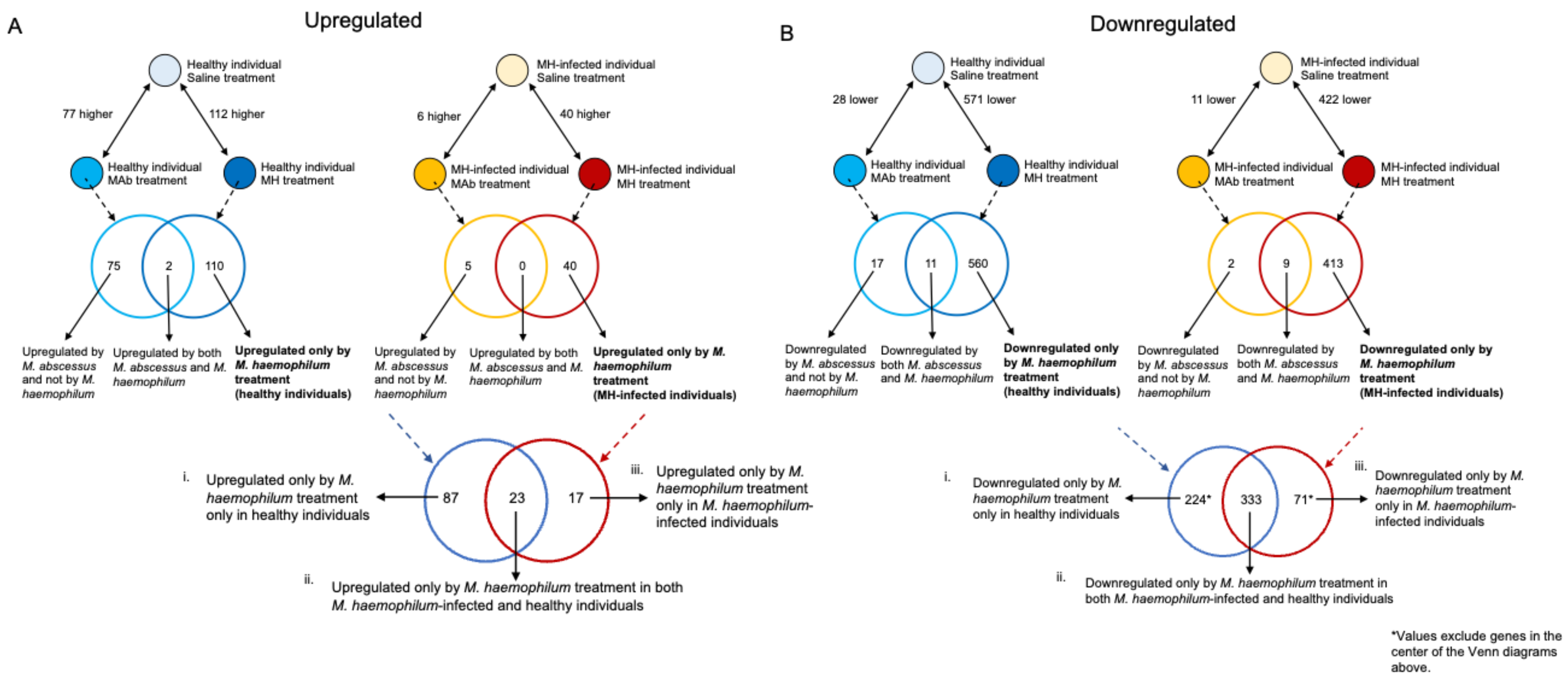

Fig. S2 Gene identification process outline. A) Identification process for genes significantly upregulated by *M. haemophilum* treatment. Paired DESeq results are overlapped, identifying genes upregulated only by *M. haemophilum* relative to saline in white blood cells from both healthy and *M. haemophilum*-infected individuals. These genes are then intersected to identify (i) 87 genes upregulated only by *M. haemophilum*, only in healthy individuals, (ii) 23 genes upregulated only by *M. haemophilum*, in both healthy and *M. haemophilum*-infected individuals, and (iii) 17 genes upregulated only by *M. haemophilum*, only in *M. haemophilum*-infected individuals; B) Identification process for genes significantly downregulated by *M. haemophilum* treatment. Paired DESeq results are overlapped, identifying genes downregulated only by *M. haemophilum* relative to saline in white blood cells from both healthy and *M. haemophilum*-infected individuals. These genes are then intersected to identify (i) 224 genes downregulated only by *M. haemophilum*, only in healthy individuals, (ii) 333 genes downregulated only by *M. haemophilum*, in both healthy and *M. haemophilum*-

infected individuals, and (iii) 71 genes downregulated only by *M. haemophilum*, only in *M. haemophilum*-infected individuals.

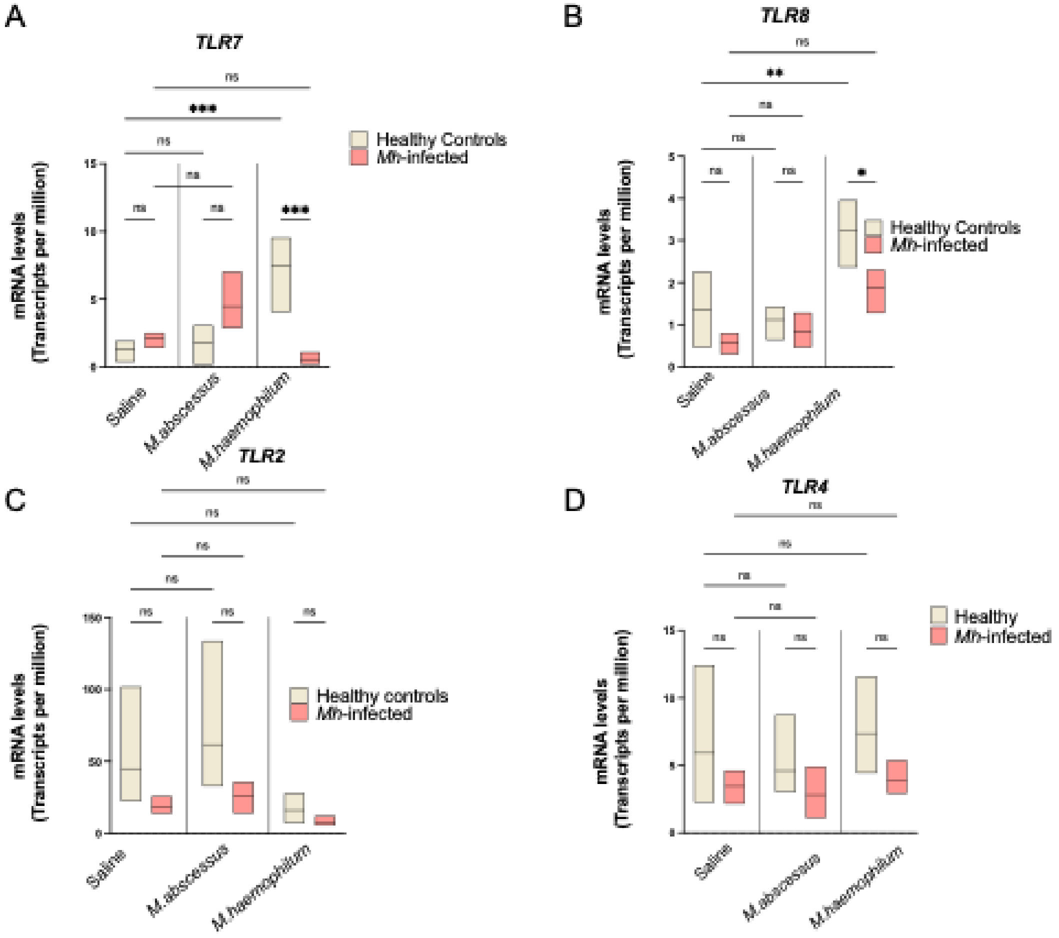

Fig.S3. *TLR* transcript levels in response to *M. abscessus* and *M. haemophilum* stimulation in HC and *Mh*-infected patients. Fragments Per Kilobase of transcript per Million mapped reads is shown as Transcripts per million. One Way ANOVA with Tukey's multiple comparison test was applied. $P < 0.05$ was considered significant. *$p<0.05$; ** $p<0.01$, *** $p < 0.001$.

Detailed Supplementary methods for Omics:

Exome Sequencing and data processing: Exome capture was performed using IDT Exome Capture Hybridization kit (xGen Lockdown Exome Panel V1). Exonic DNA was then sequenced for 300 cycles on an Illumina NovaSeq 6000 sequencer using S4 flowcells across 4 germline samples from four *Mh*-infected individuals and four HCs to an estimated coverage of 50x. Sequence read alignments were performed using bwa (1) against the human reference genome (GRCh38/hg38, version GRCh38DH), and variants were called using the Genome Analysis ToolKit (GATK) HaplotypeCaller (2). Results were annotated using Ensembl's Variant Effect Predictor (VEP)(3), with synonymous SNPs defined to include "synonymous variant", "intron variant", "5 prime UTR variant", "3 prime UTR variant", "downstream gene variant", "upstream gene variant", "stop retained variant" and "non coding transcript exon variant", and nonsynonymous SNPs were defined to include "missense variant", "frameshift variant", "stop gained", "inframe insertion", "inframe deletion", "stop lost" and "start lost". Additional annotations included (i) allele frequencies across the 125,748 exome sequences in the Genome Aggregation Database (gnomAD) v2 database (4), (ii) loss-of-function intolerance scoring using LoFtool (5), and (iii)

ensemble pathogenicity scoring spanning several algorithms and databases, using REVEL(6). The proportion of shared SNPs was calculated by dividing the number of shared SNPs between two samples and dividing by the smaller total number of high-impact SNPs between the two samples.

RNA-sequencing of ex situ stimulated donors blood cells*:* Cells from whole blood stimulation assay were cryopreserved. The cryopreserved cells were thawed and subjected to RNA preparation. RNA obtained were subjected to for bulk sequencing.

RNA-seq processing and analysis**:** Total RNA integrity was determined using Agilent Bioanalyzer or 4200 Tapestation. Library preparation was performed with 10ng of total RNA with a Bioanalyzer RIN score greater than 8.0. ds-cDNA was prepared using the SMARTer Ultra Low RNA kit for Illumina Sequencing (Takara-Clontech) per manufacturer's protocol. cDNA was fragmented using a Covaris E220 sonicator using peak incident power 18, duty factor 20%, cycles per burst 50 for 120 seconds. cDNA was blunt ended, had an A base added to the 3' ends, and then had Illumina sequencing adapters ligated to the ends. Ligated fragments were then amplified for 12-15 cycles using primers incorporating unique dual index tags. Fragments were sequenced on an Illumina NovaSeq-6000 using paired end reads extending 150 bases.

After adapter trimming using Trimmomatic v0.39 (7), sequenced RNA-seq reads were aligned to the human genome (GRCm38, Ensembl release 104(8)) using the STAR aligner v2.7.5b (9) (2-pass mode, basic). All raw RNA-Seq fastq files

are being uploaded with controlled access to European-Genome Phenome Archive. Read fragments (read pairs or single reads) were quantified per gene per sample using featureCounts v1.5.1(10).

Significantly differentially expressed genes between sample sets were identified using DESeq2 v1.4.5)(11) with default settings, and a minimum P value significance threshold of 0.05 (after False Discovery Rate [FDR(12)] correction for the number of tests). Principal components analysis also was calculated using DESeq2 output (default settings, using the top 500 most variable genes). FPKM (fragments per kilobase of gene length per million reads mapped) normalization was performed using DESeq2-normalized read counts. Z-scores of log FPKM values visualized in the heatmaps were calculated per gene and visualized using Microsoft Excel. All fragment counts, FPKM expression values, and differential expression statistics for all genes, samples and comparisons are provided in **Table S1**.

Pathway enrichment analysis among differentially expressed gene sets of interest was performed for Reactome (13) pathways using the WebGestalt (14) web server ($p \leq 0.01$ after FDR correction, minimum 3 genes per term), using a background of all protein coding genes. Pathway enrichment was extended using enrichment analysis tool Enrichr (15-17), using the default setting, available at: http://amp.pharm.mssm.edu/Enrichr. The transcription factor enrichment was carried out using Enrichr (15-17), and three different outputs including ones from “TRANSFAC and JASPAR PWMs”, “Transcription PPI”, “TF perturbations followed by Expression” compiled and reported. All considered pathways and TF were significant ($p < 0.05$).

REFERENCES

1. Li H, and Durbin R. Fast and accurate short read alignment with Burrows-Wheeler transform. *Bioinformatics*. 2009;25(14):1754-60.
2. McKenna A, Hanna M, Banks E, Sivachenko A, Cibulskis K, Kernytsky A, et al. The Genome Analysis Toolkit: a MapReduce framework for analyzing next-generation DNA sequencing data. *Genome Res*. 2010;20(9):1297-303.
3. McLaren W, Gil L, Hunt SE, Riat HS, Ritchie GR, Thormann A, et al. The Ensembl Variant Effect Predictor. *Genome Biol*. 2016;17(1):122.
4. Karczewski KJ, Francioli LC, Tiao G, Cummings BB, Alfoldi J, Wang Q, et al. The mutational constraint spectrum quantified from variation in 141,456 humans. *Nature*. 2020;581(7809):434-43.
5. Fadista J, Oskolkov N, Hansson O, and Groop L. LoFtool: a gene intolerance score based on loss-of-function variants in 60 706 individuals. *Bioinformatics*. 2017;33(4):471-4.
6. Bhaskar A, Munshi M, Khan SZ, Fatima S, Arya R, Jameel S, et al. Measuring glutathione redox potential of HIV-1-infected macrophages. *J Biol Chem*. 2015;290(2):1020-38.
7. Bolger AM, Lohse M, and Usadel B. Trimmomatic: a flexible trimmer for Illumina sequence data. *Bioinformatics*. 2014;30(15):2114-20.
8. Yates AD, Achuthan P, Akanni W, Allen J, Allen J, Alvarez-Jarreta J, et al. Ensembl 2020. *Nucleic Acids Res*. 2020;48(D1):D682-D8.
9. Dobin A, Davis CA, Schlesinger F, Drenkow J, Zaleski C, Jha S, et al. STAR: ultrafast universal RNA-seq aligner. *Bioinformatics*. 2013;29(1):15-21.

10. Liao Y, Smyth GK, and Shi W. featureCounts: an efficient general purpose program for assigning sequence reads to genomic features. *Bioinformatics*. 2014;30(7):923-30.
11. Anders S, and Huber W. Differential expression analysis for sequence count data. *Genome Biol*. 2010;11(10):R106.
12. Benjamini Y, and Hochberg Y. Controlling the False Discovery Rate: A Practical and Powerful Approach to Multiple Testing. *Journal of the Royal Statistical Society Series B (Methodological)*. 1995;57(1):289-300.
13. Fabregat A, Jupe S, Matthews L, Sidiropoulos K, Gillespie M, Garapati P, et al. The Reactome Pathway Knowledgebase. *Nucleic Acids Research*. 2018;46(D1):D649-d55.
14. Wang J, Vasaikar S, Shi Z, Greer M, and Zhang B. WebGestalt 2017: a more comprehensive, powerful, flexible and interactive gene set enrichment analysis toolkit. *Nucleic Acids Res*. 2017;45(W1):W130-W7.
15. Chen EY, Tan CM, Kou Y, Duan Q, Wang Z, Meirelles GV, et al. Enrichr: interactive and collaborative HTML5 gene list enrichment analysis tool. *BMC Bioinformatics*. 2013;14:128.
16. Kuleshov MV, Jones MR, Rouillard AD, Fernandez NF, Duan Q, Wang Z, et al. Enrichr: a comprehensive gene set enrichment analysis web server 2016 update. *Nucleic Acids Res*. 2016;44(W1):W90-7.
17. Xie Z, Bailey A, Kuleshov MV, Clarke DJB, Evangelista JE, Jenkins SL, et al. Gene Set Knowledge Discovery with Enrichr. *Curr Protoc*. 2021;1(3):e90.
